# The Decision to Vaccinate or Not during the H1N1 Pandemic: Selecting the Lesser of Two Evils?

**DOI:** 10.1371/journal.pone.0058852

**Published:** 2013-03-07

**Authors:** Andrea R. Ashbaugh, Christophe F. Herbert, Elena Saimon, Nelson Azoulay, Lening Olivera-Figueroa, Alain Brunet

**Affiliations:** 1 Research Center of the Douglas Mental Health University Institute, Montreal, Quebec, Canada; 2 Department of Psychiatry, McGill University, Montreal, Quebec, Canada; 3 Department of Psychiatry, Yale University School of Medicine, West Haven, Connecticut, United States of America; 4 Department of Psychiatry, U.S. Department of Veteran Affairs – Connecticut Healthcare System, West Haven, Connecticut, United States of America; The University of Hong Kong, Hong Kong

## Abstract

**Background:**

With the release of the H1N1 vaccine, there was much controversy surrounding its use despite strong encouragements to be vaccinated in the media. Though studies have examined factors influencing people's decision to be vaccinated, few have focused on how general beliefs about the world or where an individual gathers information might influence that decision.

**Methodology/Principal Findings:**

A cross-sectional web-based survey (*N* = 817) was conducted during the H1N1 outbreak after the vaccine was available. Variables examined included sociodemographic information, health related behaviours, specific beliefs concerning the H1N1 virus and its vaccine, as well as general beliefs, such as fear of contamination, intolerance of uncertainty, emotional states, coping behaviour, and the source of information concerning the virus. Three converging statistical methods were used to examine the associations – analysis of variance, logistic regression, and recursive partition modelling. The most consistent and strongest association was that negative beliefs about the H1N1 vaccine (e.g. fear of its side effects) was related to the decision not to be vaccinated, whereas beliefs about the dangers of the H1N1 virus was related to the decision to be vaccinated. Most notably, having very strong negative beliefs about the vaccine was a more powerful predictor than even strong beliefs about the dangers of the H1N1 virus. Furthermore, obtaining information from the Internet, as compared to more traditional sources of information (e.g., TV, newspapers) was related to the decision not to be vaccinated.

**Conclusions/Significance:**

These results are consistent with the Health Belief Model. Importantly they suggest that during future pandemics public health officials should not only discuss the dangers of the pandemic but also (i) take additional steps to reassure the public about the safety of vaccines and (ii) monitor the information disseminated over the Internet rather than strictly relying on the more traditional mass media.

## Introduction

In April 2009 an outbreak of a new Influenza called H1N1 occurred in Mexico. It spread rapidly around the world and it was reported in the news that a number of healthy people died from it. In June 2009, the World Health Organization (WHO) declared the H1N1 Influenza outbreak a ‘pandemic’, meaning that it had spread to several continents, if not the whole world [1]. The pandemic was characterized by two waves, the first occurring in the Spring 2009, and the second occurring in the Fall 2009 [2] and by December 2009, at the time the current study was conducted, the second wave was beginning to wane [3]. In total, in Canada there were 33 509 cases identified, and 428 deaths from H1N1 [4]. By the mid-fall 2009 a vaccine against the H1N1 Influenza was available in Canada and individuals were encouraged to get the vaccine by the public health authorities [5]. The media provided extensive coverage on the H1N1 pandemic and its vaccine. The information was mostly in favour of the vaccine, but some sources of information strongly criticized the safety of the vaccine [6]. Information was readily available on various sites on the Internet, including both official health agencies [7] and unofficial health forums [e.g.,8]. The request in search engines like Google for ‘H1N1’ was so popular that it was possible to conduct search-term surveillance and Web-based mapping. Online social networks (e.g. Facebook, Twitter) also became a source of information about the H1N1 virus and the vaccine [9]. By the end of the pandemic it was estimated that in Canada 41% of individuals were vaccinated [10]. Though this rate is higher than vaccination rates for the seasonal flu vaccine [11], [12], it remains very much under the target of 100% of Canadians that the government had selected [13]. It is of interest to note that the majority of individuals still chose not to be vaccinated in spite of the fact that (i) health services are free in Canada, (ii) that the vaccine was available on the work premises of many people, (iii) and that important financial resources were invested in order to convince the population via the mass media to get the vaccine.

### Factors Affecting Vaccination Decisions

Several studies have examined psychosocial predictors of vaccination against influenza [11], [14], [15] and against the H1N1 virus specifically [15]–[20]. In general, these studies found that factors predicting vaccination status/intention included perceived severity of the H1N1 virus and fear of adverse side effects or safety of the vaccine. Other factors that also predicted vaccination intention included greater trust in the government [19], [21], being non-Caucasian [18], and being older [18], [19]. However, there are several limitations to these studies. Many studies focused only on specific subgroups of individuals such as pregnant women [17] or health care workers [20]. Additionally, many studies relied on general questions about perceptions of the H1N1 virus [15], [18], [19]. Also, very few studies have examined other health related behaviours people may have engaged in during the pandemic, and the one that did, failed to examine how the engagement in such behaviours relates to the decision to be vaccinated [15]. It may be, for example, that individuals who engage in other health behaviours, such as hand washing, are more likely to choose to be vaccinated. Other limitations include a small sample size, or the use of statistical methods that may not capture complicated relationships, such as nonlinear relationships. Finally, we could find only one study that examined the influence of traditional media (e.g. TV, Newspapers) versus the Internet in the decision to get the vaccine against the H1N1 virus. Fabry and colleagues found that pregnant women were more likely to be vaccinated if they consulted official government websites, and less likely to be vaccinated if they consulted mainstream websites, but found no effect for other types of media, including official government leaflets, or mainstream television [17]. The Internet is a unique from other media sources by the fact that anyone can easily post their opinion about any given topic (e.g. pro or cons vaccine) and have it read by millions people. Furthermore, research suggests that 58% of U.S. Internet users report being influenced by online information in treatment decision [22], though the information may be false or incomplete. Additionally, research examining the type of information available on the Internet, concerning vaccines in general, find that a higher proportion of ‘hits’ resulting from web searches for terms such as ‘vaccination’ are anti-vaccination in nature [23]. It is therefore important to assess the relative impact of Internet use on H1N1 vaccination intention.

The Health Belief Model (HBM) model suggests that an individual will decide to engage in a given behaviour aimed at preventing or treating a disease based upon their beliefs about the perceived threat of the disease and beliefs about the benefits and risks of the target behaviour. Two recent reviews examined predictors of H1N1 vaccination intention and action and found, consistent with the HBM model, that beliefs about the perceived threat of the disease and the benefits and risks of being vaccinated were important predictors [24], [25]. Additionally they identified several other factors that predicted behaviour change including several demographic variables (being older, male, more educated, and non-Caucasian).

All studies reported above focused on specific beliefs related to the H1N1 virus, and ignored individual differences in general beliefs and characteristics that may influence the decision making process, such as the ability to tolerate uncertainty, general fears of contamination, coping styles, and current emotional state. A few studies have examined the relationship between emotional states and the use of preventative behaviours, such as mask wearing and hand washing (excluding vaccination), during the H1N1 pandemic, finding that higher levels of state anxiety predict greater engagement in such behaviours [26], [27]. With regards to the H1N1 vaccine, Savas found that individuals Turkish healthcare workers with higher levels of state anxiety was found among Turkish healthcare workers who believed the vaccination was unsafe [28]. Additionally, one study demonstrated that individuals with somatoform or anxiety problems, particularly Obsessive Compulsive Disorder, a disorder in which fear of contamination features prominently, exhibited an exacerbation of their psychiatric symptoms as a result of fears related to H1N1 [29]. To the best of our knowledge, no study has examined how individual differences in intolerance of uncertainty or coping style may influence health behaviour, including the decision to be vaccinated, in relationship to the H1N1 virus. However, both of these variables have been found to influence other health related behaviours. For example, individuals reporting high levels of intolerance of uncertainty engage in more health monitoring [30] and individuals with avoidant coping styles are less likely to follow doctors' specific recommendations [31]. These studies all suggest that emotional states and individual differences in some beliefs may influence health related behaviour. It may be that these are also factors that influence an individual's decision to be vaccinated.

The purpose of the current study is to examine factors related to the decision to be vaccinated in a predominantly Canadian sample. Factors examined include beliefs related to the H1N1 virus and vaccine that correspond to the HBM model, as well as other health behaviours individuals may have used in an effort to prevent contracting the H1N1 virus. A second objective of this study is to examine where individuals obtain their information (e.g. TV, Internet, discussion with loved-ones) about the H1N1 virus and vaccine, and the impact these sources of information may have on decision making.

Furthermore, in an effort to improve the reliability of responses we assessed beliefs about the H1N1 virus and vaccine, and health behaviours people engaged in during the pandemic by creating multi-item questionnaires to assess the constructs we were interested in evaluating (one for H1N1 beliefs and one for behaviours), rather than assessing these constructs using a single question. Additionally, we also examined the degree to which general fears of contamination, the inability to tolerate uncertainty, coping style, current emotional state and important information sources were related to the decision to be vaccinated against the H1N1 virus. We predicted, consistent with the HBM model, that stronger beliefs about the seriousness of the H1N1 virus and safety of its vaccine, as well as greater engagement in other preventative behaviours would predict the choice to be vaccinated. As little research has examined the relationship between fear of contamination, intolerance of uncertainty, emotional states, source of information, and the decision to be vaccinated no specific predictions about these variables were made.

Finally, we opted to use a number of converging statistical methods as a means to examine the associations between the variables of interest: analysis of variance (ANOVA), logistic regression, and the Classification And Regression Tree (CART) approach. Most previous studies have relied upon the use of logistic regression or ANOVA to examine predictors of vaccination status [17]–[20], as logistics regression reports the odds of being vaccinated for a given variable and ANOVA is helpful in identifying whether individuals who intend to be vaccinated are different on a given variable from individuals who do not intend to be vaccinated. Though both methods provide valuable information concerning the relationship between vaccination behaviour and other variables, the interpretation of complex interactions between continuous variables can be difficult using these methods [32]. CART can uncover complex and non-linear interactions between variables that would not be discovered using more traditional regression techniques [32], [33]. We report results using ANOVA and logistic regression to enable comparison with previous studies and CART to help understand the complex interaction between factors that predict the intention to be vaccinated against the H1N1 virus.

## Results

### Sociodemographic Characteristics

Of participants that completed at least 90% of the questions (*n* = 817), 94% indicated that they resided in Canada and 6% resided elsewhere. Forty percent of participants indicated that they intended to or did receive the vaccine (Pro-vaccine), 54% indicated that they did not intend to receive the vaccine (Anti-vaccine) and 6% reported that they were undecided (Undecided). Participants were on average about 35 years of age (range 18–65), about 70% were female, and the majority were Caucasian. Most participants indicated that they had received at least some college education, indicating that this was a fairly educated sample. Table 1 presents other sociodemographic data for this sample of participants divided into those who intended to receive the vaccine (Pro-vaccine), did not intend to receive the vaccine (Anti-vaccine) and were undecided (Undecided). There were no significant sociodemographic differences between the groups except concerning ethnicity, the percentage of participants indicating they were a member of a high-risk group in terms of contracting the H1N1 virus, and the percentage of individuals working as a health professional.

**Table 1:** Sociodemographics of participants by vaccination intention.

Category	Subcategory	Pro-Vaccine	Anti-Vaccine	Undecided
*N*		327	437	52
Age *M (SD)*		36.83 (13.64)	35.00 (12.46)	36.46 (13.65)
Female (%)		72.7	70.1	75.0
Education (%)				
	Less than Highschool	1.8	2.1	1.9
	Highschool	6.5	10.8	13.5
	Some college	32.0	36.8	34.6
	College	43.1	39.3	38.5
	Graduate School	16.6	11.0	11.5
Ethnicity (%)^a^				
	Caucasian	82.1	84.5	65.4
	African American	0	.9	1.9
	Hispanic	.6	1.6	7.7
	Asian	9.0	5.8	11.5
	Aboriginal	4.3	2.8	0
	Middle Eastern	1.9	1.4	5.8
	Other	2.2	3.0	7.7
High Risk (%)* b		41.5	23.3	23.1
Health Professional (%)^c^		17.2	7.8	8.0

* At risk groups were defined as individuals indicating that they are currently pregnant, have a chronic medical condition, are immune suppressed, or have children at home under the age of 5.

a χ^2^ = 36.52, *p*<.001.

b χ^2^ = 30.22, *p*<.001.

c χ^2^ = 16.70, *p*<.001.

### Health Behaviour Model Variables

Exploratory factor analyses with varimax rotation were conducted on the Pandemic Behaviour Questionnaire and Vaccination Questionnaire – which were constructed specifically for this study- to extract relevant factors using the scree plot and with an eigen value above 1. For the Pandemic Behaviour Questionnaire, the initial factor analysis yielded a five-factor solution, explaining 52.37% of the variance. The first factor (15 items) assessed preventative behaviours (Prevention). Sample items from the Prevention subscale include “I avoid touching my mouth and nose” and “I clean my hands frequently with soap and water”. The second factor (6 items) assessed avoidance behaviour (Avoidance). Sample items from the Avoidance subscale include “I avoid spending time in crowded settings” and “I avoid leaving the house unless absolutely necessary”. The third factor (3 items) included items referring to mask wearing (Mask Wearing), such as “I wear a mask even if I am not sick.” The fourth factor (2 items) included items assessing personal care (Personal Care), such as “I have been trying to eat a well-balanced diet.” The final factor (3 items) assessed preparatory behaviour (Preparation), such “I have seen a doctor to get a prescription for Tamiflu even though I am not sick.” Cronbach's alpha for each scale were 0.91, 0.75, 0.70, 0.70, and 0.27 respectively. As the final factor, Preparation, had an unacceptable reliability, and removing items from that factor did not improve the reliability, this factor was not included in further analyses. After removal of these items, a four-factor solution was obtained accounting for 52.45% of the variance. Subscale scores were calculated by taking the mean of all items contributing to that subscale, thus scores for each of the four subscales ranged from 0 to 4, with higher values indicating greater engagement in that type of behaviour.

Table 2 presents the means (*M*) and standard deviations (*SD*) for the four factors of the Pandemic Behaviour Questionnaire by Vaccination Intention groups. There was a significant difference between the Vaccination Intention groups for Prevention, Avoidance, and Mask Wearing, but not Personal Care. The Anti-Vaccine group scored significantly lower on Prevention than the Pro-Vaccine and the Undecided groups. The Undecided group scored significantly higher on Avoidance than the Pro-Vaccine and the Anti-Vaccine groups. The Anti-Vaccine group reported engaging in significantly less mask wearing than the Pro-Vaccine and Undecided groups.

**Table 2:** Score on the Pandemic Behaviour Questionnaire and Vaccination Questionnaire by vaccination intention groups.

Category	Subcategory	Pro-Vaccine	Anti-Vaccine	Undecided	*F* a
Pandemic Behaviour Questionnaire					
	Prevention *M (SD)*	2.55^x^ (.66)	2.36^y^ (.82)	2.71^x^ (.64)	9.13**
	Avoidance *M (SD)*	1.68^x^ (.72)	1.64^x^ (.81)	2.00^y^ (.76)	5.03**
	Mask Wearing *M (SD)*	.60^x^ (.85)	.33^y^ (.65)	.57^x^ (.96)	13.56**
	Personal Care *M (SD)*	2.67 (.79)	2.81 (.94)	2.91 (.72)	2.91
Vaccination Questionnaire					
	H1N1 Beliefs *M (SD)*	3.09^x^ (.68)	1.69^y^ (.71)	2.53^z^ (.64)	375.40**
	Negative Vaccination Beliefs (*M SD)*	1.18^x^ (.62)	2.45^y^ (.76)	1.85^z^ (.66)	312.03**

*Note.* Means with differing superscripts (e.g., ^x, y, z^) indicate a significant difference at the .05 level or more.

a df  = 2, 813.

** *p*<.001.

For the Vaccination Questionnaire, factor analysis yielded a five-factor solution accounting for 54.89% of the variance, however, the last two factors contained only two and one items respectively with no readily interpretable theme for the two item factor. The factor analysis was recalculated without these items yielding a three-factor solution accounting for 51.29% of the variance. As Cronbach's alpha for the final factor, containing 4 items, was unacceptable (α = 0.50), this factor was not retained for further analyses. When the factor analysis was recalculated without these four items it yielded a two-factor solution accounting for 53.01% of the variance. The first factor (8 items) assessed negative beliefs about the vaccine (Negative Vaccination Beliefs). The scale included 2 items that were reverse scored. Sample items include “The use of thermasil (mercury preservative) in the H1N1 vaccine can have serious consequences,” and “The benefits of the vaccine outweigh the risks” (reverse scored). The second factor (6 items) assessed beliefs about the dangers of the H1N1 virus (H1N1 Beliefs). The scale included 1 item that was reverse scored. Items included, “I am concerned about getting H1N1 because healthy people died from it” and “I want to protect myself from the serious consequences of H1N1”. Cronbach's alphas for the two subscales were 0.86, and 0.77 respectively. Subscale scores were calculated by taking the mean of all items contributing to that score, thus scores for each of the two subscales ranged from 0 to 4.

Table 2 presents the means and standard deviations for Vaccination Questionnaire subscale scores for each Vaccination Intention group. There were significant differences between the Vaccination Intention groups for both H1N1 Beliefs and Negative Vaccination Beliefs. The Pro-Vaccine group reported the strongest beliefs about the danger of the H1N1 virus, and the Anti-Vaccine group reported the weakest beliefs about the danger of the H1N1 virus. Conversely, the Pro-Vaccine group reported the weakest beliefs about the dangers of the H1N1 vaccine and the Anti-Vaccine group reported the strongest beliefs about the dangers of the H1N1 vaccine.

### Affective, Coping, and Cognitive Variables

Table 3 displays the means and standard deviations for the contamination subscale of the Vancouver Obsessive Compulsive Inventory (VOCI contamination) [34], the uncertainty subscale of the Obsessive Beliefs Questionnaire (OBQ uncertainty) [35], the anxiety, depression and stress subscales of the Depression Anxiety Stress Scale (DASS) [36], and approach, diversion, and resignation/withdrawal subscales of the Brief Approach Avoidance Coping Questionnaire (Coping) [37]. There were significant differences between the Vaccination Intention groups on the following variables: VOCI contamination, DASS anxiety, OBQ uncertainty, Coping diversion and Coping resignation/withdrawal. Differences between the groups approached but did not reach statistical significance for DASS depression and stress.

**Table 3:** Affective, cognitive and coping variable scores by vaccination intention groups.

Category	Subcategory	Pro-Vaccine	Anti-Vaccine	Undecided	*F* a
DASS Subscales					
	Anxiety *M (SD)*	6.77^ y^ (8.14)	5.62 ^x^ (7.34)	8.50^ x^ (8.98)	4.33*
	Stress *M (SD)*	11.65 (10.00)	10.65^ x^ (9.72)	13.91^ y^ (10.94)	2.94†
	Depression *M (SD)*	8.69 (10.40)	7.55^ x^ (9.65)	10.74^ y^ (11.12)	2.94†
VOCI Contamination *M (SD)*		11.31^x^ (9.81)	8.82^y^ (8.66)	13.12^x^ (13.44)	9.29**
OBQ Uncertainty *M (SD)*		46.83^x^ (12.79)	44.30^y^ (13.42)	44.72 (15.86)	3.41*
Coping					
	Approach *M (SD)*	22.00 (3.92)	22.53 (3.82)	22.05 (3.46)	1.88
	Diversion *M (SD)*	8.94^x^ (2.30)	8.46^y^ (2.45)	9.12 (1.86)	4.91**
	Resignation/Withdrawal *M (SD)*	6.90^ x^ (2.65)	6.48^ y^ (2.61)	7.15 (2.66)	3.26*

*Note.* Means with differing superscripts (e.g., ^x, y, z^) are significantly different from each other at the .05 level or more.

a *df*  = 2, 813.

† *p*<.06, * *p*<.05, ** *p*<.01.

Follow-up pairwise comparisons were calculated. The Undecided group reported significantly more stress, depression, and anxiety compared to the Anti-Vaccine group. It also reported significantly more anxiety compared to the Pro-Vaccine group. The Anti-Vaccine group reported less fear of contamination, whereas the Pro-Vaccine group reported greater intolerance for uncertainty. The Anti-Vaccine also reported less fear of contamination compared to the Undecided group. The Pro-Vaccine group reported significantly more use of diversion and resignation/withdrawal as coping tools compared to the Anti-Vaccine group.

### Influential Sources of Information

There was a significant difference in the type of information participants indicated was most influential in their decision on whether to be vaccinated, *χ^2^* (*df*  = 8)  = 48.50, *p*<.001. Though it is clear from Table 4 that most individuals based their decision upon discussions with family, friends and co-workers, nearly 30% participants in the Anti-Vaccine group indicated that the Internet was the most influential source of information (compared to less than 15% of participants in the other two groups), and nearly 30% of participants in the Undecided group indicated that the television and printed media were influential (compared to 15–20% in the other two groups).

**Table 4:** Most influential information source for the decision to be vaccinated.

Category	Pro-Vaccine (%)	Anti-Vaccine (%)	Undecided (%)
Television	10.3	9.2	25.5
Radio	3.4	1.9	2.0
Newspaper/Magazine	9.6	7.5	17.6
Internet	13.9	30.4	13.7
Family/Friends/Co-workers	62.5	51.1	41.2

*Note:* Cells represent the percentage of participants in each group who endorsed the item as being most influential in their decision.

### Logistic Regression

Having identified variables that distinguished between those who intended to receive the vaccine, those who did not intend to receive the vaccine, and those who were undecided about getting the vaccine, we sought to identify which constellation of these variables best distinguished these groups from each other using two methods, multinomial regression and classification and regression tree (CART) analyses. Those variables that were significant or nearly significant (i.e., *p*<.10, two-tailed test) in the initial ANOVAs presented above were used in subsequent analyses. Variables included in the analyses were therefore ethnicity, high risk status, working as a health care professional, subscales of the Pandemic Behaviour Questionnaire and Vaccination Questionnaire, VOCI Contamination, OBQ Uncertainty, DASS stress, depression, and anxiety, and coping diversion, and withdrawal/resignation. The reference category for analyses was the Pro-Vaccine group. Initial analysis using multinomial regression indicated that some predictor variables should be excluded or some categories merged. Given the smaller number of participants in the Undecided group, analyses were rerun merging the Undecided group with the Anti-Vaccine group using logistic regression since the goal of the current analyses were to identify factors that can help identify those individuals who may not choose to be vaccinated. The reference category for the logistic regression remained the Pro-Vaccine group. Anaylses were also conducted eliminating the Undecided group rather than combining them with those who did not intend to be vaccinated. Results remained nearly identical with the exception that those reporting high risk status were now less likely to be vaccinated.

Analyses revealed that the model fit the data well, χ ^2^ (*df*  = 25)  = 630.60, *p*<.0001, −2 log likelihood  = 423.75. The Hosmer-and-Lemeshow fit statistic indicated that the final model was nearly a good fit with the data, χ^2^ (8, *N* = 816)  = 15.41, *p* = .052. The classification accuracy of the final model was 89.5% (87.9% Pro-Vaccine – sensitivity; 90.6% Anti-Vaccine/Undecided – specificity). Table 5 presents the Odds Ratios (OR) for all variables.

**Table 5:** Odds ratio for vaccination intention (Yes or No/Undecided) (*N* = 816).

Category	Subcategory	Wald	OR	95% CI	
Ethnicity^a^		6.18			
High Risk Group^b^		16.46**	3.20	1.82	5.61
Health Professional^c^		6.65**	2.98	1.30	6.83
H1N1 Beliefs		73.37**	6.87	4.39	10.56
Negative Vaccination Beliefs		69.37**	.15	.10	.24
Avoidance		2.35	.71	.46	1.10
Prevention		1.04	.79	.51	1.24
Mask Wearing		5.05*	1.55	1.06	2.27
Personal Care		7.78**	.63	.45	.87
DASS- Anxiety		.00	1.00	.96	1.05
DASS- Stress		.00	1.00	.96	1.04
DASS – Depression		2.68	.97	.94	1.01
VOCI- Contamination		8.23**	1.05	1.02	1.09
OBQ- Uncertainty		1.47	1.02	.99	1.04
Coping – Diversion		.13	1.02	.91	1.14
Coping – Resignation/Withdrawal		.05	1.01	.90	1.15
Information Source^d^		16.34**			
	Television	7.02**	.31	.13	.73
	Radio	1.00	.47	.11	2.05
	Newspaper/Magazine	5.40*	.36	.14	.85
	Internet	9.65**	.31	.15	.65

*Note*: Odds ratios (OR) that have a 95% confidence interval that includes the value 1.0 are not significant at the .05 level.

a Reference category for ethnicity is Caucasian.

b Reference category for High Risk Group is no.

c Reference category for Health Professional is no.

d Reference category for Preferred Information Source is Discussions with Friends and Family.

* *p*<.10, * *p*<.05; ** p<.01.

Variables that significantly predicted group membership included High Risk Status, being a Health Professional, Negative Vaccination Beliefs, H1N1 Beliefs, Avoidance, Mask Wearing, Personal Care, VOCI Contamination, and Information Source. Individuals who were identified as being at high risk or indicated that they were employed as a health professional were roughly three times more likely to choose to be vaccinated. Those who reported greater concern about the H1N1 virus as measured by the Vaccination Questionnaire H1N1 Beliefs subscale were nearly seven times more likely to choose to be vaccinated. Those who reported engaging in more mask wearing as measured by the Pandemic Behaviour Questionnaire Mask Wearing subscale were 1.5 times more likely to choose to be vaccinated, and those who reported greater fear of contamination as measured by the VOCI contamination subscale were 1.05 times more likely to choose to be vaccinated. Conversely, those who reported greater concern about vaccinations as measured by the Vaccination Questionnaire Negative Vaccination Beliefs subscale were six times less likely to choose to be vaccinated. Those who indicated that printed media, the television, or Internet were influential sources of information as compared to discussions with family or friends were roughly three times less likely to be vaccinated.

### CART Decision Tree

**Figure 1:**
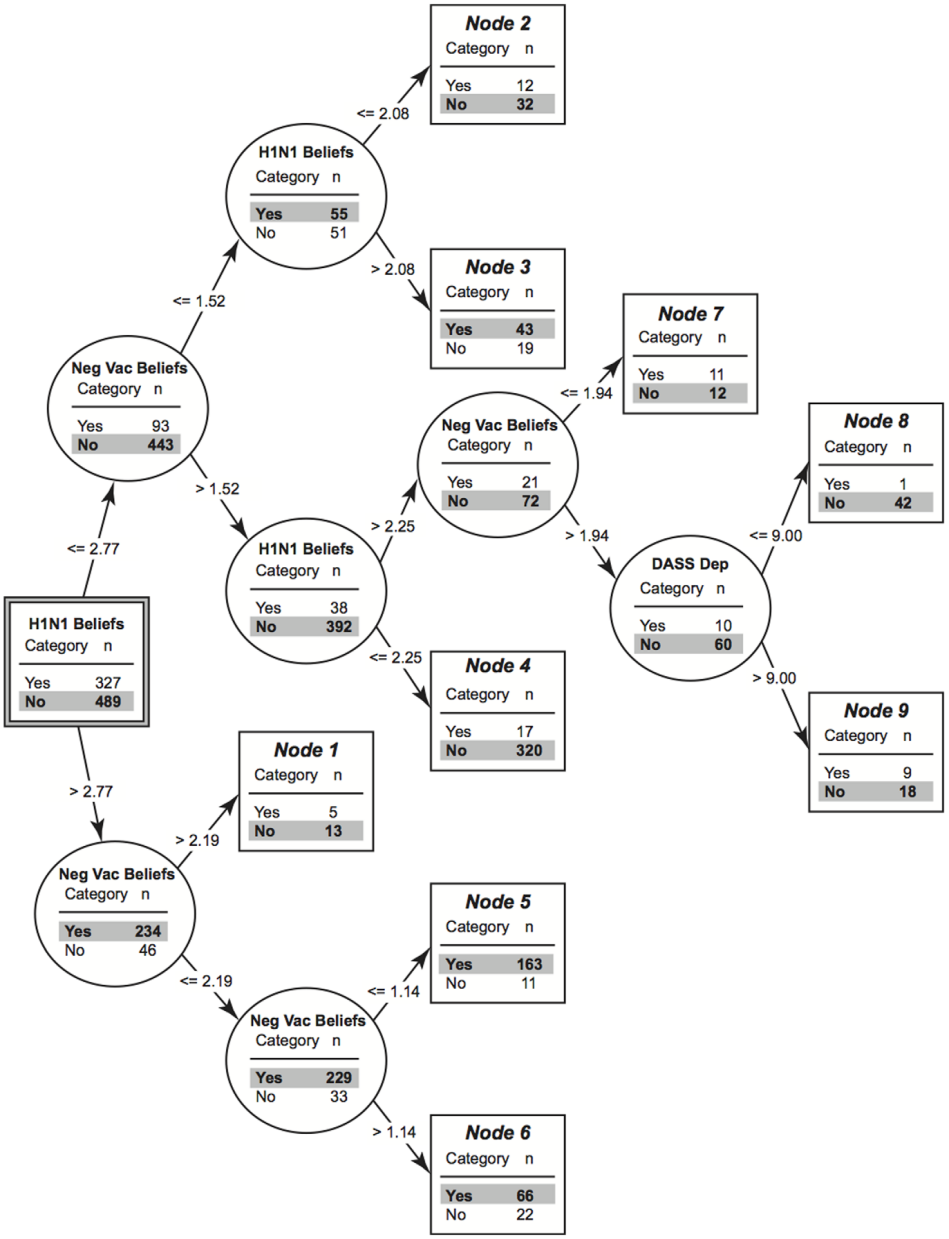
Classification and regression tree (CART) model depicting variables discriminating those who were (Yes) and were not vaccinated or were undecided (No). *Note*: The square box with double lines represents the starting node, all other square boxes represent terminal nodes. Circular boxes represent nodes containing branches. The title contained within circular boxes indicates the measure used to divide participants into the next node with the dividing values indicated along the arrows. *Neg Vac Beliefs*  =  Negative Vaccination Beliefs.

Figure 1 depicts the results of classification model developed using CART analysis. The model created 5 levels of classification. As the tree generated was less complex than expected, the decision was made to not prune the tree. The model generated correctly classified 83.2% of participants who chose to be vaccinated and 89.4% of participants who chose not to be vaccinated or were undecided. To verify the accuracy of the model generated we reran the CART analysis with different minimum *n*s for parent and child nodes. Overall the model remained relatively stable with these changes and thus we concluded that the model generated was accurate.

Overall nine ending nodes were generated. Participants were first divided based upon their scores on the Vaccination Questionnaire H1N1 Beliefs. Participants scoring at or below 2.77 were separated from participants scoring above 2.77. The model generated suggests that it is primarily a combination of beliefs between two evils: the dangers of the H1N1 virus in conjunction with beliefs about the dangers of being vaccinated that ultimately determines whether one intended to be vaccinated. Participants reporting moderate to strong beliefs about the dangers of the H1N1 virus as evidenced by generally high scores on the H1N1 Beliefs subscale of the Vaccination Questionnaire and moderate to weak beliefs about the dangers of being vaccinated were more likely to indicate that they intended to be vaccinated (Terminal nodes 3, 5, and 6). Individuals reporting strong beliefs about the dangers of being vaccinated as measured by the Negative Vaccination Beliefs subscale of the Vaccination Questionnaire regardless of the beliefs they hold about the H1N1 virus were more likely to indicated that they did not intend to be vaccinated (Terminal nodes 1, 4, 9, 8). Individuals who do not have strong beliefs about either being vaccinated or the H1N1 virus also indicated that they did not intend to be vaccinated (Terminal Node 2) whereas holding moderate beliefs about both the dangers of being vaccinated and the dangers of the H1N1 virus results in an even split with regards to the number of individuals intending to be vaccinated and not intending to be vaccinated (Terminal Node 7).

## Discussion

The current study attempted to identify factors that were related to an individual's decision to be vaccinated during the H1N1 pandemic. Most previous studies examined only specific beliefs related to the H1N1 virus. Not only did the current study examine those specific beliefs, but also Behaviours engaged in during the pandemic, as well as general beliefs that may be related to the decision making process, such as fear of contamination, intolerance of uncertainty, coping style, and certain emotional states. The source of information about the virus and vaccine and how this information was related to the decision to be vaccinated was also examined. Finally, previous studies have examined these beliefs in highly specific at-risk samples, such as pregnant women. This current study examined these beliefs in a more general convenience sample of Internet users.

Overall, our study replicated and extended the results of previous studies [15]–[20], [38] under improved methodological conditions: Individuals who intended to be vaccinated reported stronger beliefs about the dangers of H1N1 and weaker beliefs about the dangers of the vaccine. They also tended to report greater intolerance of uncertainty, higher levels of anxiety, and the use of more avoidant coping strategies than those who were not vaccinated. These findings suggest that those who intended to be vaccinated believed that the H1N1 virus was dangerous. Their decreased ability to tolerate uncertainty combined with higher levels of anxiety may have affected the degree to which they engaged in safety behaviours (e.g., getting vaccinated) to prevent getting sick. Individuals who did not intend to be vaccinated reported engaging in less preventative behavioural strategies or mask wearing, and reported less fear of contamination than those who were undecided or chose to be vaccinated. These findings suggest that this group may be less concerned about the dangers of H1N1, which is consistent with the fact that they scored lower than those who were vaccinated on beliefs about the dangers of the H1N1 virus. Importantly, these individuals were also more likely to report that the Internet was an influential source of information compared to the other two groups. Finally, individuals who were undecided about the vaccine reported more use of avoidance strategies to prevent H1N1, and higher levels of stress and depression. These individuals may be engaging in avoidance behaviour because they have not been vaccinated yet. Their higher levels of stress and depression may hinder their ability to make decisions concerning their health. These individuals also reported that print media and the television were influential sources of information compared to the other two groups.

The main sociodemographic variables identified as predictors of the intention to be vaccinated were being a member of a high-risk group, and being a health professional. We found that individuals identified as being in a high-risk group were more likely to be vaccinated. This is consistent with findings from previous research [18], [24], [39]. In contrast, the fact that health professionals in our sample were more likely to be vaccinated is inconsistent with Virseda and colleague's [20] study, which found that only 16.5% of health care workers were vaccinated against H1N1, whereas 49.7% were vaccinated against the seasonal influenza. It is possible that these results reflect regional differences in the acceptability of the vaccine, as the sample in the current study was primarily North American, whereas Virseda et al.'s sample was from a hospital in Spain.

Gender and age were unrelated to vaccination intention. This is consistent with several studies assessing the decision to be vaccinated against the H1N1 virus [16], [20], though some studies have found that older individuals were more willing to be vaccinated [18], [19]. It is notable that methods of recruitment varied greatly across these different studies, ranging from recruitment primarily via facebook in the current study to recruitment in hospitals [20] to online research panels [18]. These different recruitment methods may be one possible explanation for the diversity of findings. For example, in the current study older adults may have been underrepresented, which may have affected our ability to detect age effects.

In addition to replicating results concerning the importance of beliefs related to the virus and its vaccine in the decision to be vaccinated, we also identified several other variables, including some specific health related Behaviours, general beliefs, and different methods of obtaining information about the virus that differentiated those who intended to be vaccinated from those who did not intend to be vaccinated. Concerning specific behaviours individuals engaged in during the H1N1 pandemic, we found that individuals who intended to or were contemplating being vaccinated were more likely to engage in preventative behaviours, including mask wearing; and that those who were contemplating being vaccinated were more likely to engage in avoidance behaviours. However, though there were differences between the groups, only the mask wearing and personal care variables influenced the odds of intending to be vaccinated. Self-reported mask wearing increased the odds of intending to be vaccinated, whereas greater personal care (e.g., eating a well balanced meal) was associated with lower odds of intending to be vaccinated. It may be that those who employed active and direct strategies aimed at preventing the disease (e.g., hand washing, mask wearing) were more likely to intend to be vaccinated, whereas individuals who engaged in more general strategies that promote good health (e.g., personal care) are less likely to be vaccinated, perhaps because they believe that a healthy lifestyle reduces the risk of contracting the virus. The tendency of individuals who were undecided about taking the vaccine to engage in more avoidant behaviours may reflect efforts to protect oneself against the virus until they reached a decision. As we could find no other study that examined the relationship between being vaccinated and other preventative behaviours, it is difficult to draw conclusions concerning these mixed results and any explanations of the current findings remain speculative. The current findings nonetheless are consistent with other research that suggests that regular vaccination use (another preventative behaviour) predicts future vaccination [12], [18], [21], [40]. The relationship between other types of behaviour aimed at avoiding the H1N1 and/or other viruses and vaccination warrant further research and could provide insight into certain subgroups of individuals (e.g., people engaged in healthy lifestyles) that may benefit from more direct encouragement to vaccinate.

Concerning general beliefs that may predict vaccination behaviour, we found that individuals who reported more fear of contamination and more intolerance of uncertainty were more likely to be vaccinated. However, only fear of contamination increased the odds of being vaccinated, though to a very small degree. It may be that these general beliefs influence specific beliefs about the H1N1 virus and its vaccine, rather than vaccination behaviour directly. Future research should assess this possibility using mediation models. Additionally, other beliefs that were not assessed in the current study may have influence on vaccination behaviour. For example, Setbon and colleagues [19] found that the belief in conspiracy theories decreased the odds of being vaccinated against the H1N1 virus.

Concerning the relationship between various emotions and the decision to be vaccinated, we found that individuals who were undecided at the time of the survey reported more anxiety, stress, and depression than participants from the other two groups. However, these emotional states failed to influence the odds of being vaccinated, though this was likely due to the fact that the undecided group was combined with the anti-vaccine group for these analyses, and therefore differences between these two groups were obfuscated. Previous research suggests that greater psychological distress increased engagement in preventative behaviours early on during the H1N1 pandemic, before the vaccine was available [26], [41], [42]. These somewhat inconsistent findings that greater emotional distress predicts higher engagement in some preventative behaviours, but indecision to engage in others (i.e., being vaccinated) warrant further investigation.

Another unique contribution of this study was to examine what source of information (e.g., T.V., Internet, etc.) was influential in helping individuals make the decision on whether to be vaccinated. More than for other flus, there was an increase of media coverage about the influenza and the vaccine in 2009, particularly on the Web. As research shows, people are increasingly using the Internet to search information about health [43] and are influenced by it [44]. Individuals who chose not to be vaccinated tended to cite the Internet as an influential source, whereas those who were undecided tended to cite print media and the television as influential sources. Our results suggest that health policy makers should attend carefully to information available on the Internet, do more to increase the visibility of official websites, and be more present on the social networking websites (e.g. Facebook, Twitter).

Though we did attempt to examine whether individuals who were undecided about being vaccinated were different from individuals who intended to or did not intend be vaccinated, unfortunately due to the small sample size of this group were unable to conduct all planned analyses including this group. It is notable that the undecided group did appear to differ from the other groups on a number of variables. Individuals who were undecided were more ethnically diverse, reported engaging in more avoidance behaviours, reported higher levels of stress and depression, and indicated that television and newspapers were influential sources of information. They also tended to report beliefs about the severity of the H1N1 virus and the dangers of the H1N1 vaccine that fell somewhere in the middle between those who intended to and those who did not intend to be vaccinated. They resembled individuals who did not intend to be vaccinated with regards to the percentage who reported belonging to a high risk group or being a health professional and their degree of anxiety and resembled individuals who intended to be vaccinated concerning the degree of preventative behaviours, including mask wearing, they reported engaging in and the degree to which the feared contamination. It is notable that the odds of belonging to the group that intended to be vaccinated did not change whether the undecided group was included with those who did not intend to be vaccinated or excluded from analyses with the exception that the odds were reversed for belonging to a high risk group. That is, belonging to a high risk group increased the odds of being vaccinated when the undecided group was combined with those who did not intend to be vaccinated, but decreased the odds of being vaccinated when the undecided group was excluded from analyses. We could find only one other study that explicitly examined individuals who were undecided about whether to be vaccinated against the H1N1 virus [45]. In contrast to our study, the study conducted by Arda and colleagues was restricted to health care professionals, and included a larger proportion of the sample who were undecided. In contrast to our study where age was related to vaccination intention, they found that being younger was associated with being undecided about whether to be vaccinated. Those who were undecided were also more concerned about the side effects of the vaccine. These two studies highlight some potential unique factors associated with vaccination indecision and suggest that future research should further explore reasons for their indecision [45].

Though individuals differed on a number of variables described above, it is notable that the strongest and most consistent predictor of the decision to be vaccinated across the three selected statistical approaches used were beliefs about the dangers H1N1 virus and negative beliefs about the H1N1 vaccine. This is consistent with the HBM model and extends results from several other studies examining the prediction of vaccination behaviour during the H1N1 pandemic [16]–[21] and in general [40], [46].

We extend these findings by demonstrating that having very strong beliefs that the vaccine was dangerous appears to override beliefs about the dangers of the H1N1 virus in deciding whether or not to be vaccinated among. We further extended this finding by showing the role played by the Internet.

There are some limitations to the current study that warrant mention. First, as this study design was cross-sectional in nature it is not possible to establish a causal relationship between beliefs about the H1N1 influenza and vaccine, other non-H1N1 related beliefs, and the decision to be vaccinated. For example, based upon the results obtained in this study we are unable to determine if beliefs about the vaccine and virus lead to vaccination intentions or if vaccinations intentions lead to beliefs about the vaccine and virus that might be predicted by biases such as the confirmation bias. Longitudinal research is required to establish directionality of these relationships. Longitudinal research would also be useful for examining how general beliefs concerning contamination and affective states impact the development of cognitions concerning a specific disease, such as the H1N1 influenza. A second limitation concerns sampling, recruitment and generalizability. The sampling population was restricted to those having access to the Internet and using the English language to browse. It is notable that a large proportion of our sample was female and highly educated. This sampling bias may have affected results particularly concerning demographic characteristics as previous research has found that men [25] and those with more education [24] were more likely to be vaccinated. Therefore results, particularly concerning demographic factors should be interpreted with caution. That being said, one should note that 84.3% of Canadians (where the majority of are participants were recruited from) are connected to the Internet [47]. In order to achieve a truly representative sample other recruitment strategies should be used in future research. Finally, due to the small number of undecided individuals we were forced to group them with those who chose not to be vaccinated for the logistic regression, and CART analysis. The unfortunate result of this is that some interesting differences observed between the undecided group and the other groups during the initial analyses were potentially obfuscated in the logistic and CART analyses. As individuals who were undecided appear to differ on important variables, notably emotional distress, and the use of avoidance behaviour, further research examining this subgroup would be beneficial, particularly since they may be more open to being vaccinated than those who clearly stated they would not be vaccinated.

The current study examined the impact of not only specific beliefs about the H1N1 virus and its vaccine, but also the impact of general beliefs about the world, specific health related behaviours engaged in, emotional states, and how individuals obtained information about the virus on the decision to be vaccinated. As the H1N1 virus was different than traditional flus (pandemic declared by W.H.O., number of deaths, and the very large world media coverage), our results seems to be more specific to H1N1-like flus. In summary, results suggests that the most important factors in determining if an individual is likely to be vaccinated are beliefs about the dangers of the H1N1 influenza and its vaccine. Though people who were vaccinated differed from those who were undecided or did not choose to be vaccinated on a number of different variables, it was beliefs about the H1N1 influenza and its vaccine that most consistently distinguished the groups across three different methods of analyses. Results indicate that very strong beliefs about the dangers of being vaccinated are especially powerful. This suggests that focusing not only on educating the public about the seriousness of the Influenza, but equally on educating the public about the safety of a vaccine may be helpful in increasing the rates of vaccination. Furthermore, results suggest that the Internet may have been a particularly salient source of negative information about the vaccine. Internet is a powerful and uncontrolled source of information where people can debate about the safety or dangerosity of the vaccine and the influenza. This suggests that government agencies should increase their presence and credibility on the Internet and social networking sites.

## Materials and Methods

### Ethics Statement

This research was approved by the Institutional Review Board (IRB) of the Douglas Mental Health University Institute, in Montreal, Quebec and was conducted according to the Declaration of Helsinki. Prior to beginning the study participants were asked to read the consent form and provide informed consent by clicking on a button that stated “I have read this form and have decided to participate.”

### Design

The study consisted of an online survey that was accessible on the Internet between December 10, 2009 and January 7, 2010.

### Participants

Participants were recruited via English language advertisements posted on the *Facebook* pages of individuals whose Internet Protocol (IP) address indicated that they resided in Canada, the United States, or Europe, as well as ads on free websites including the Douglas Mental Health University Institute website, and info-trauma.org. The ad appeared approximately 25 867 455 times between December 10 2009 and January 12 2010 and was clicked on 7802 times. One thousand fifty-two individuals began the survey and 817 completed at least 90% of the items in the study. Remaining missing data was imputed using maximum likelihood estimation. The demographic characteristics of participants appear in Table 1. Participants who completed the study were eligible to have their name entered in a draw to win one of five small Internet redeemable gifts certificates ($25–100 value). Participants could also remain totally anonymous (with the exception of their IP address).

### Measures

In addition to basic demographic information, participants were asked to complete self-report questionnaires concerning health related behaviours in response to the H1N1 pandemic, current cognitions and emotions and coping style. Each questionnaire appeared on a separate web page. Participants clicked next at the end of each page to move on to the next questionnaire.

*Vaccination Intention* was assessed by asking participants “Do you plan on taking the vaccine?” Participants had the option of responding “Yes” “No” or “Undecided,” and were instructed to select yes if they had already been vaccinated.

#### High Risk Status

Participants were asked to indicate if they were a member of the following groups that were classified as being high risk: currently pregnant, have a chronic medical condition, are immune suppressed, or have children at home under the age of 5.

#### Influential Sources of Information

Participants were asked to indicate which of the following sources of information were most influential in their decision about whether to be vaccinated: television, radio, newspaper/magazine, Internet, discussions with family and friends.

*Pandemic Behaviour Questionnaire* asked participants to rate how frequently they have engaged in a series of behaviours in the past month using a 5-point Likert scale ranging from 0 (Never) to 5 (Always). Item development was based on recommendations to reduce the risk of contracting H1N1 influenza posted by the governments of Canada, United States, Australia, and the United Kingdom, as well as the WHO. Sample items include “I avoid touching my mouth and nose” and “I clean my hands frequently with soap and water.” Additional items to assess more extreme behaviours that were not necessarily recommended by governing agencies were also created, such as “I have avoided leaving the house unless absolutely necessary.”

*Vaccination Questionnaire* asked participants to rate using a 5-point Likert scale from 0 (Not at all true) to 5 (Extremely true) how strongly they agree with various reasons for their decision on whether or not to be vaccinated. Sample items include “I don't think the virus is as dangerous as it is portrayed,” “I am afraid of the side effects,” ”I don't want to pass the H1N1 virus to my children and relatives in case I contract it,” and “Because I follow the advice of professionals or people I trust.”

#### Uncertainty Subscale, Obsessive Beliefs Questionnaire [35]

The OBQ is a 88-item self-report questionnaire that assesses cognitions related to obsessive compulsive disorder obsessive compulsive disorder. The Uncertainty subscale assesses the degree to which individuals believe that uncertainty is intolerable, with higher scores indicating greater intolerance of uncertainty. The scale has demonstrated excellent internal consistency, test-retest reliability, and convergent and divergent validity in clinical samples [35].

Contamination Subscale, *Vancouver Obsessional Compulsive Inventory* [34] The VOCI is a 55-item self-report questionnaire assessing a variety of symptoms associated with obsessive compulsive disorder. The Contamination subscale assesses an individual's preoccupation with cleanliness and avoidance of germs and other perceived sources of contamination. The scale has demonstrated excellent internal consistency, test-retest reliability, and convergent and divergent validity in clinical and non-clinical samples [34], [48].

#### Depression Anxiety Stress Scale 21

[36] is a 21-item self-report questionnaire with three subscales assessing symptoms of depression (e.g., sadness, worthlessness), anxiety (e.g., trembling, faintness), and stress (e.g., tension, irritability). This short form of the DASS has demonstrated excellent internal consistency and concurrent validity in clinical and non-clinical samples [49].

*Brief Approach Avoidance Coping Questionnaire* [37] is a 12-item questionnaire assessing the dimensions of approach versus avoidance coping. The scale reflects one dichotomous dimension of approach versus avoidance coping, with two sub-categories of avoidance coping – resignation/withdrawal and diversion. The questionnaire has demonstrated adequate reliability and moderate convergent validity [37].

#### Statistical Analyses

Items on the Pandemic Behaviour Questionnaire and Vaccination Questionnaire were first submitted to an exploratory factor analysis to identify factors for subsequent analyses. In order to examine what factors distinguished individuals who were vaccinated from those who were not or were undecided about being vaccinated three separate analyses were conducted. First, factors of the Pandemic Behaviour Questionnaire and Vaccination Questionnaire that were determined to have acceptable reliability, as well as scores on the affective, cognitive measures, and sociodemographic variables were compared across Vaccination Intention groups (Pro-Vaccine, Anti-Vaccine, Undecided) using one-way between-participant ANOVAs and *χ^2^* . Variables that were found to be significantly related to Vaccination Intention were used as predictor variables in a multinomial logistic regression and recursive partitioning analysis. For the multinomial logistic regression, all items that significantly differentiated the Vaccination Intention groups were included in the model. Vaccination Intention was used as the predictor. The goodness of fit for the model was obtained using the Hosmer-and-Lemeshow goodness-of-fit statistic [50]. In this statistic low *χ^2^* and high *p*-values indicate an acceptable fit. To examine the contribution of each variable Odds Ratios (OR), and the 95% confidence intervals (CI) surrounding the OR are presented.

For the recursive partitioning analysis, the CART method was used. In CART a decision tree is created by partitioning the data into binary groups that maximize the homogeneity within each group based upon Gini criterion. These groups are split again until further splitting no longer decrease impurity by a factor greater than .0001. We specified that parent nodes have a minimum *n* of 25 and child nodes have a minimum *n* of 10 in order for additional nodes to be generated. To validate the tree-generated cross validation, in which the sample is divided into subsamples excluding 10 participants per sample, was employed. The tree presented represents the average of all trees generated. The advantage of using recursive partitioning analysis over other methods, such as logistic regression, is that it allows for the examination of non-linear relationship [51]. All analyses were conducted using SPSS, version 19.
